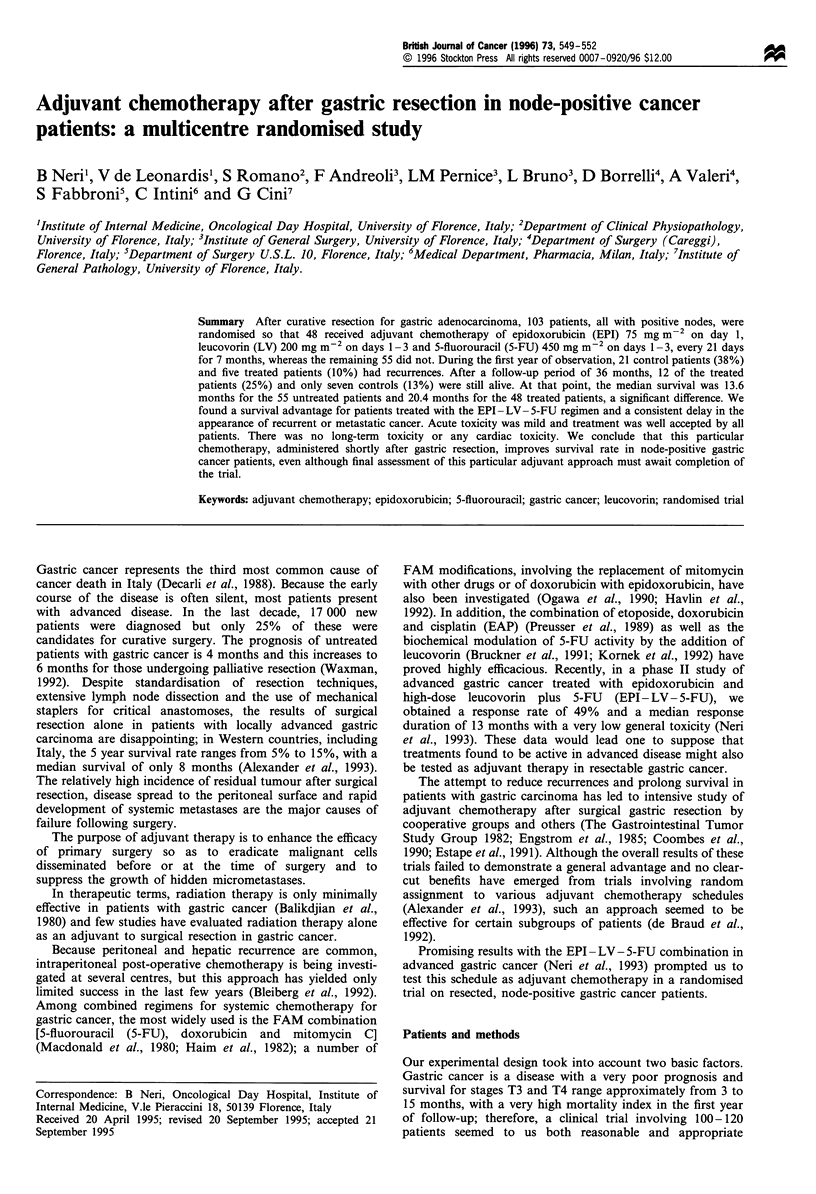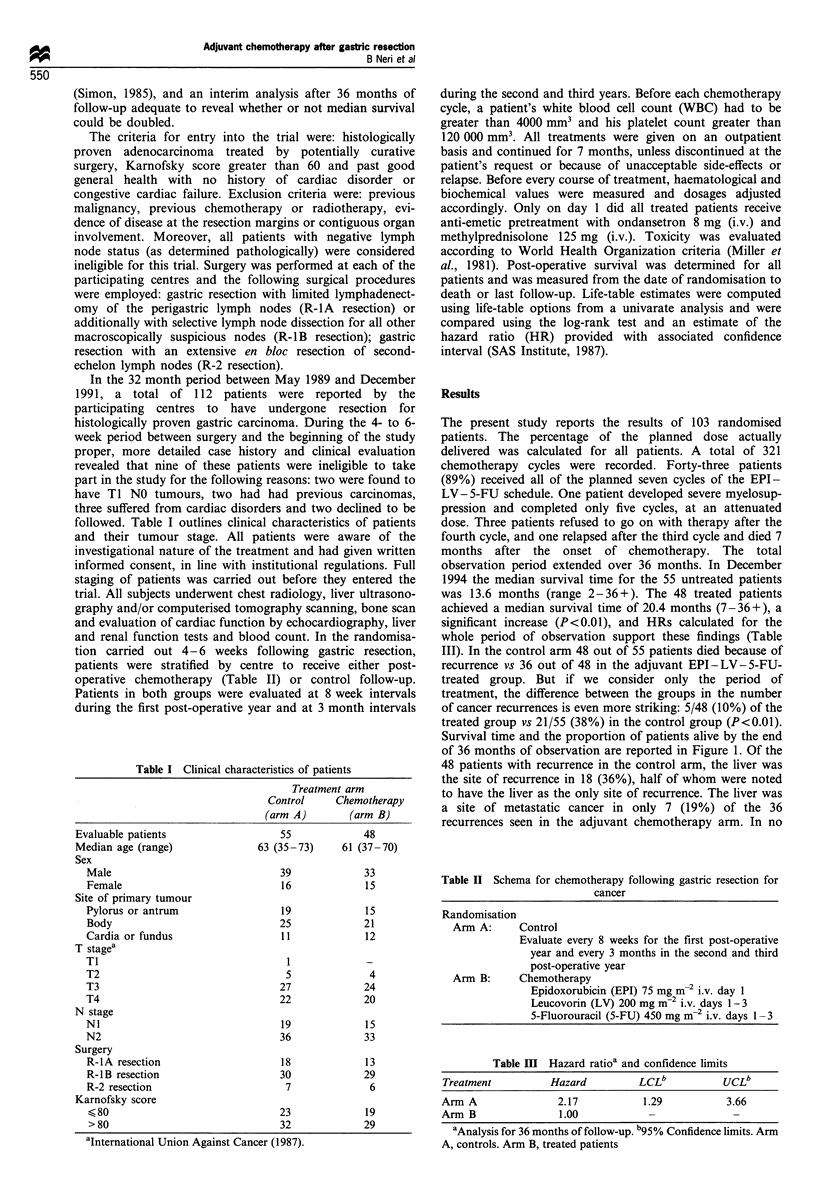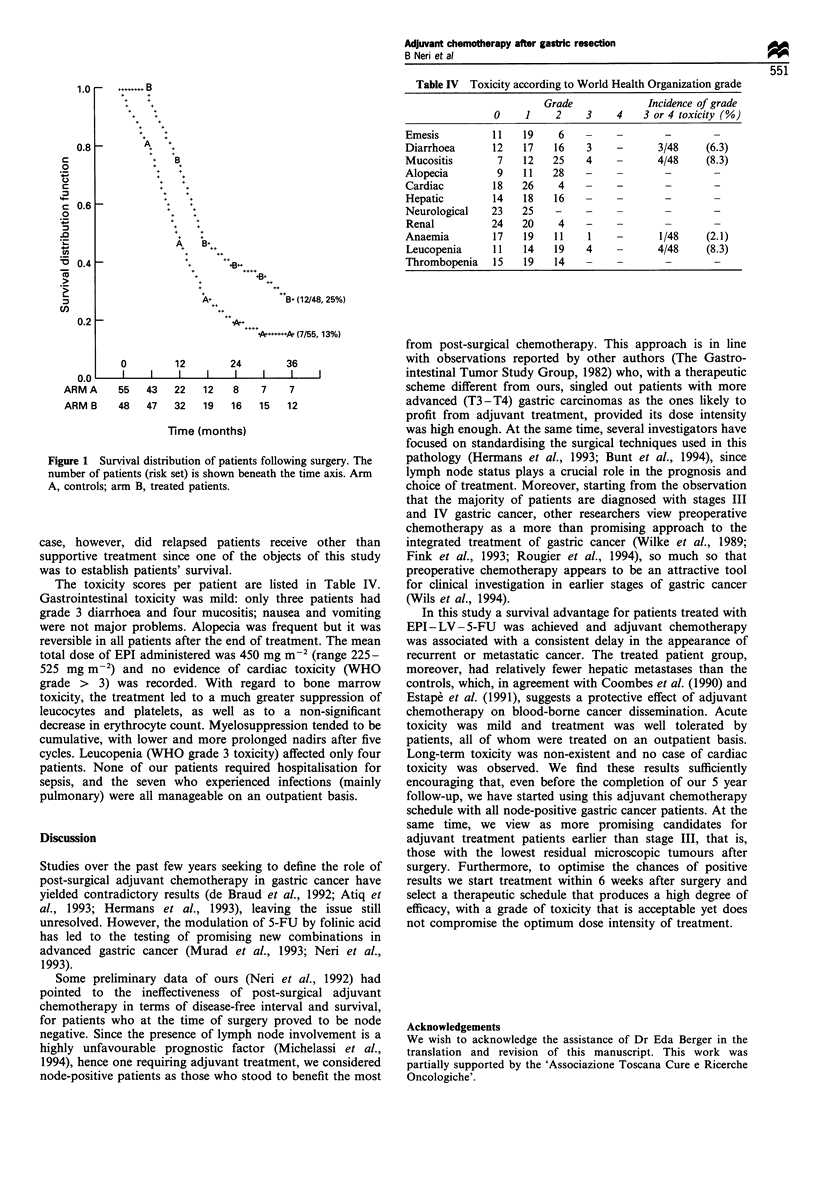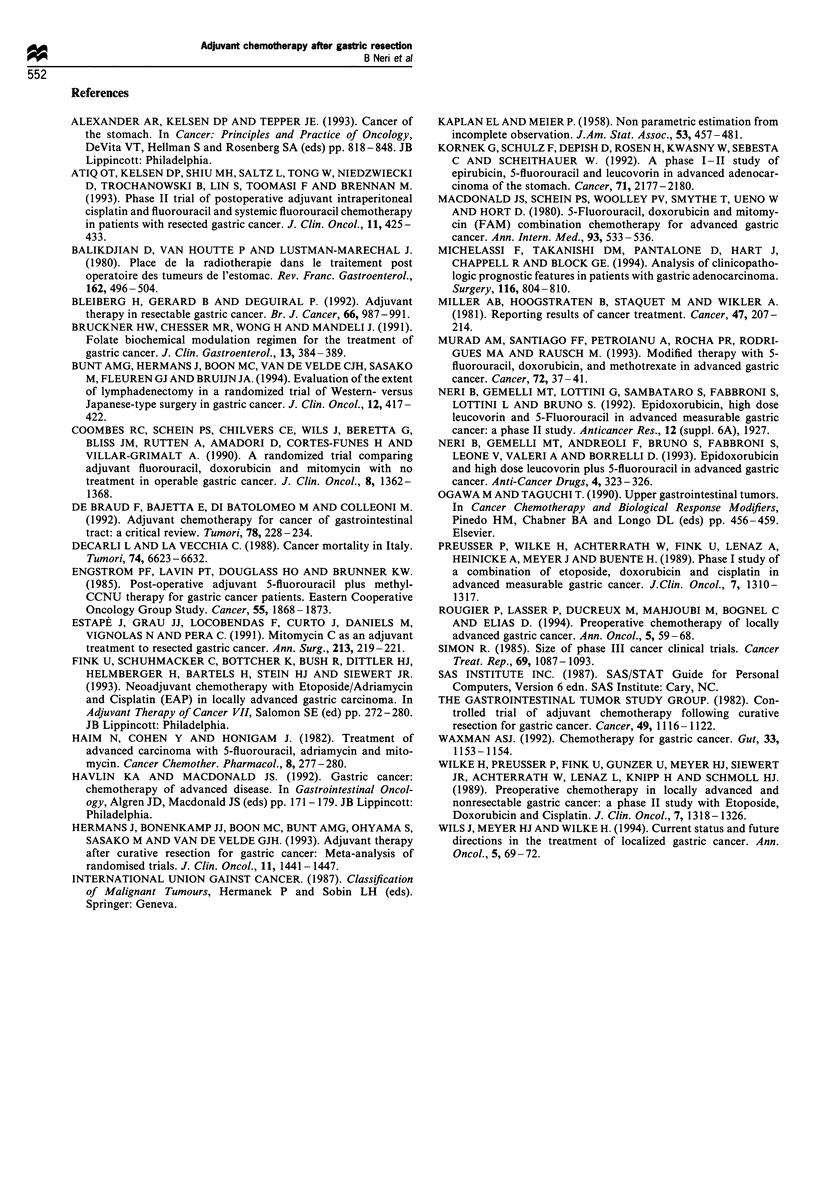# Adjuvant chemotherapy after gastric resection in node-positive cancer patients: a multicentre randomised study.

**DOI:** 10.1038/bjc.1996.95

**Published:** 1996-02

**Authors:** B. Neri, V. de Leonardis, S. Romano, F. Andreoli, L. M. Pernice, L. Bruno, D. Borrelli, A. Valeri, S. Fabbroni, C. Intini, G. Cini

**Affiliations:** Institute of Internal Medicine, Oncological Day Hospital, University of Florence, Italy.

## Abstract

After curative resection for gastric adenocarcinoma, 103 patients, all with positive nodes, were randomised so that 48 received adjuvant chemotherapy of epidoxorubicin (EPI) 75 mg m-2 on day 1, leucovorin (LV) 200 mg m-2 on days 1-3 and 5-fluorouracil (5-FU) 450 mg m-2 on days 1-3, every 21 days for 7 months, whereas the remaining 55 did not. During the first year of observation, 21 control patients (38%) and five treated patients had recurrences. After a follow-up period of 36 months, 12 of the treated patients (25%) and only seven controls (13%) were still alive. At that point, the median survival was 13.6 months for the 55 untreated patients and 20.4 months for the 48 treated patients, a significant difference. We found a survival advantage for patients treated with the EPI-LV-5-FU regimen and a consistent delay in the appearance of recurrent or metastatic cancer. Acute toxicity was mild and treatment was well accepted by all patients. There was no long-term toxicity or any cardiac toxicity. We conclude that this particular chemotherapy, administered shortly after gastric resection, improves survival rate in node-positive gastric cancer patients, even although final assessment of this particular adjuvant approach must await completion of the trial.


British Journal of Cancer (1996) 73, 549-552
© 1996 Stockton Press All rights reserved 0007-0920/96 $12.00

### Adjuvant chemotherapy after gastric resection in node-positive cancer patients: a multicentre randomised study

B Neri[1], V de Leonardis[1], S Romano[2], F Andreoli[3], LM Pernice[3], L Bruno[3], D Borrelli[4], A Valeri[4], S Fabbroni[5], C Intini[6] and G Cini[7]

[1]*Institute of Internal Medicine, Oncological Day Hospital, University of Florence, Italy;* [2]*Department of Clinical Physiopathology, University of Florence, Italy;* [3]*Institute of General Surgery, University of Florence, Italy;* [4]*Department of Surgery (Careggi), Florence, Italy;* [5]*Department of Surgery U.S.L. 10, Florence, Italy;* [6]*Medical Department, Pharmacia, Milan, Italy;* [7]*Institute of General Pathology, University of Florence, Italy.*

**Summary** After curative resection for gastric adenocarcinoma, 103 patients, all with positive nodes, were randomised so that 48 received adjuvant chemotherapy of epidoxorubicin (EPI) 75 mg $m^{-2}$ on day 1, leucovorin (LV) 200 mg $m^{-2}$ on days 1-3 and 5-fluorouracil (5-FU) 450 mg $m^{-2}$ on days 1-3, every 21 days for 7 months, whereas the remaining 55 did not. During the first year of observation, 21 control patients (38%) and five treated patients (10%) had recurrences. After a follow-up period of 36 months, 12 of the treated patients (25%) and only seven controls (13%) were still alive. At that point, the median survival was 13.6 months for the 55 untreated patients and 20.4 months for the 48 treated patients, a significant difference. We found a survival advantage for patients treated with the EPI-LV-5-FU regimen and a consistent delay in the appearance of recurrent or metastatic cancer. Acute toxicity was mild and treatment was well accepted by all patients. There was no long-term toxicity or any cardiac toxicity. We conclude that this particular chemotherapy, administered shortly after gastric resection, improves survival rate in node-positive gastric cancer patients, even although final assessment of this particular adjuvant approach must await completion of the trial.

**Keywords:** adjuvant chemotherapy; epidoxorubicin; 5-fluorouracil; gastric cancer; leucovorin; randomised trial

Gastric cancer represents the third most common cause of cancer death in Italy (Decarli *et al.*, 1988). Because the early course of the disease is often silent, most patients present with advanced disease. In the last decade, 17 000 new patients were diagnosed but only 25% of these were candidates for curative surgery. The prognosis of untreated patients with gastric cancer is 4 months and this increases to 6 months for those undergoing palliative resection (Waxman, 1992). Despite standardisation of resection techniques, extensive lymph node dissection and the use of mechanical staplers for critical anastomoses, the results of surgical resection alone in patients with locally advanced gastric carcinoma are disappointing; in Western countries, including Italy, the 5 year survival rate ranges from 5% to 15%, with a median survival of only 8 months (Alexander *et al.*, 1993). The relatively high incidence of residual tumour after surgical resection, disease spread to the peritoneal surface and rapid development of systemic metastases are the major causes of failure following surgery.

The purpose of adjuvant therapy is to enhance the efficacy of primary surgery so as to eradicate malignant cells disseminated before or at the time of surgery and to suppress the growth of hidden micrometastases.

In therapeutic terms, radiation therapy is only minimally effective in patients with gastric cancer (Balikdjian *et al.*, 1980) and few studies have evaluated radiation therapy alone as an adjuvant to surgical resection in gastric cancer.

Because peritoneal and hepatic recurrence are common, intraperitoneal post-operative chemotherapy is being investigated at several centres, but this approach has yielded only limited success in the last few years (Bleiberg *et al.*, 1992). Among combined regimens for systemic chemotherapy for gastric cancer, the most widely used is the FAM combination [5-fluorouracil (5-FU), doxorubicin and mitomycin C] (Macdonald *et al.*, 1980; Haim *et al.*, 1982); a number of FAM modifications, involving the replacement of mitomycin with other drugs or of doxorubicin with epidoxorubicin, have also been investigated (Ogawa *et al.*, 1990; Havlin *et al.*, 1992). In addition, the combination of etoposide, doxorubicin and cisplatin (EAP) (Preusser *et al.*, 1989) as well as the biochemical modulation of 5-FU activity by the addition of leucovorin (Bruckner *et al.*, 1991; Kornek *et al.*, 1992) have proved highly efficacious. Recently, in a phase II study of advanced gastric cancer treated with epidoxorubicin and high-dose leucovorin plus 5-FU (EPI-LV-5-FU), we obtained a response rate of 49% and a median response duration of 13 months with a very low general toxicity (Neri *et al.*, 1993). These data would lead one to suppose that treatments found to be active in advanced disease might also be tested as adjuvant therapy in resectable gastric cancer.

The attempt to reduce recurrences and prolong survival in patients with gastric carcinoma has led to intensive study of adjuvant chemotherapy after surgical gastric resection by cooperative groups and others (The Gastrointestinal Tumor Study Group 1982; Engstrom *et al.*, 1985; Coombes *et al.*, 1990; Estape *et al.*, 1991). Although the overall results of these trials failed to demonstrate a general advantage and no clear-cut benefits have emerged from trials involving random assignment to various adjuvant chemotherapy schedules (Alexander *et al.*, 1993), such an approach seemed to be effective for certain subgroups of patients (de Braud *et al.*, 1992).

Promising results with the EPI-LV-5-FU combination in advanced gastric cancer (Neri *et al.*, 1993) prompted us to test this schedule as adjuvant chemotherapy in a randomised trial on resected, node-positive gastric cancer patients.

### Patients and methods

Our experimental design took into account two basic factors. Gastric cancer is a disease with a very poor prognosis and survival for stages T3 and T4 range approximately from 3 to 15 months, with a very high mortality index in the first year of follow-up; therefore, a clinical trial involving 100-120 patients seemed to us both reasonable and appropriate

Correspondence: B Neri, Oncological Day Hospital, Institute of Internal Medicine, V.le Pieraccini 18, 50139 Florence, Italy
Received 20 April 1995; revised 20 September 1995; accepted 21 September 1995

(Simon, 1985), and an interim analysis after 36 months of follow-up adequate to reveal whether or not median survival could be doubled.

The criteria for entry into the trial were: histologically proven adenocarcinoma treated by potentially curative surgery, Karnofsky score greater than 60 and past good general health with no history of cardiac disorder or congestive cardiac failure. Exclusion criteria were: previous malignancy, previous chemotherapy or radiotherapy, evidence of disease at the resection margins or contiguous organ involvement. Moreover, all patients with negative lymph node status (as determined pathologically) were considered ineligible for this trial. Surgery was performed at each of the participating centres and the following surgical procedures were employed: gastric resection with limited lymphadenectomy of the perigastric lymph nodes (R-1A resection) or additionally with selective lymph node dissection for all other macroscopically suspicious nodes (R-1B resection); gastric resection with an extensive *en bloc* resection of second-echelon lymph nodes (R-2 resection).

In the 32 month period between May 1989 and December 1991, a total of 112 patients were reported by the participating centres to have undergone resection for histologically proven gastric carcinoma. During the 4- to 6-week period between surgery and the beginning of the study proper, more detailed case history and clinical evaluation revealed that nine of these patients were ineligible to take part in the study for the following reasons: two were found to have T1 N0 tumours, two had had previous carcinomas, three suffered from cardiac disorders and two declined to be followed. Table I outlines clinical characteristics of patients and their tumour stage. All patients were aware of the investigational nature of the treatment and had given written informed consent, in line with institutional regulations. Full staging of patients was carried out before they entered the trial. All subjects underwent chest radiology, liver ultrasonography and/or computerised tomography scanning, bone scan and evaluation of cardiac function by echocardiography, liver and renal function tests and blood count. In the randomisation carried out 4–6 weeks following gastric resection, patients were stratified by centre to receive either post-operative chemotherapy (Table II) or control follow-up. Patients in both groups were evaluated at 8 week intervals during the first post-operative year and at 3 month intervals during the second and third years. Before each chemotherapy cycle, a patient's white blood cell count (WBC) had to be greater than 4000 $mm^3$ and his platelet count greater than 120 000 $mm^3$. All treatments were given on an outpatient basis and continued for 7 months, unless discontinued at the patient's request or because of unacceptable side-effects or relapse. Before every course of treatment, haematological and biochemical values were measured and dosages adjusted accordingly. Only on day 1 did all treated patients receive anti-emetic pretreatment with ondansetron 8 mg (i.v.) and methylprednisolone 125 mg (i.v.). Toxicity was evaluated according to World Health Organization criteria (Miller *et al.*, 1981). Post-operative survival was determined for all patients and was measured from the date of randomisation to death or last follow-up. Life-table estimates were computed using life-table options from a univarate analysis and were compared using the log-rank test and an estimate of the hazard ratio (HR) provided with associated confidence interval (SAS Institute, 1987).

**Table I** Clinical characteristics of patients

| | Treatment arm Control (arm A) | Chemotherapy (arm B) |
|---|---|---|
| Evaluable patients | 55 | 48 |
| Median age (range) | 63 (35–73) | 61 (37–70) |
| Sex | | |
| Male | 39 | 33 |
| Female | 16 | 15 |
| Site of primary tumour | | |
| Pylorus or antrum | 19 | 15 |
| Body | 25 | 21 |
| Cardia or fundus | 11 | 12 |
| T stage[a] | | |
| T1 | 1 | – |
| T2 | 5 | 4 |
| T3 | 27 | 24 |
| T4 | 22 | 20 |
| N stage | | |
| N1 | 19 | 15 |
| N2 | 36 | 33 |
| Surgery | | |
| R-1A resection | 18 | 13 |
| R-1B resection | 30 | 29 |
| R-2 resection | 7 | 6 |
| Karnofsky score | | |
| $\leqslant$80 | 23 | 19 |
| >80 | 32 | 29 |

[a]International Union Against Cancer (1987).

### Results

The present study reports the results of 103 randomised patients. The percentage of the planned dose actually delivered was calculated for all patients. A total of 321 chemotherapy cycles were recorded. Forty-three patients (89%) received all of the planned seven cycles of the EPI–LV–5-FU schedule. One patient developed severe myelosuppression and completed only five cycles, at an attenuated dose. Three patients refused to go on with therapy after the fourth cycle, and one relapsed after the third cycle and died 7 months after the onset of chemotherapy. The total observation period extended over 36 months. In December 1994 the median survival time for the 55 untreated patients was 13.6 months (range 2–36+). The 48 treated patients achieved a median survival time of 20.4 months (7–36+), a significant increase ($P<0.01$), and HRs calculated for the whole period of observation support these findings (Table III). In the control arm 48 out of 55 patients died because of recurrence *vs* 36 out of 48 in the adjuvant EPI–LV–5-FU-treated group. But if we consider only the period of treatment, the difference between the groups in the number of cancer recurrences is even more striking: 5/48 (10%) of the treated group *vs* 21/55 (38%) in the control group ($P<0.01$). Survival time and the proportion of patients alive by the end of 36 months of observation are reported in Figure 1. Of the 48 patients with recurrence in the control arm, the liver was the site of recurrence in 18 (36%), half of whom were noted to have the liver as the only site of recurrence. The liver was a site of metastatic cancer in only 7 (19%) of the 36 recurrences seen in the adjuvant chemotherapy arm. In no

**Table II** Schema for chemotherapy following gastric resection for cancer

| | |
|---|---|
| Randomisation | |
| Arm A: | Control |
| | Evaluate every 8 weeks for the first post-operative year and every 3 months in the second and third post-operative year |
| Arm B: | Chemotherapy |
| | Epidoxorubicin (EPI) 75 mg $m^{-2}$ i.v. day 1 |
| | Leucovorin (LV) 200 mg $m^{-2}$ i.v. days 1–3 |
| | 5-Fluorouracil (5-FU) 450 mg $m^{-2}$ i.v. days 1–3 |

**Table III** Hazard ratio[a] and confidence limits

| *Treatment* | *Hazard* | *LCL*[b] | *UCL*[b] |
|---|---|---|---|
| Arm A | 2.17 | 1.29 | 3.66 |
| Arm B | 1.00 | – | – |

[a]Analysis for 36 months of follow-up. [b]95% Confidence limits. Arm A, controls. Arm B, treated patients

**Figure 1** Survival distribution of patients following surgery. The number of patients (risk set) is shown beneath the time axis. Arm A, controls; arm B, treated patients.

case, however, did relapsed patients receive other than supportive treatment since one of the objects of this study was to establish patients' survival.

The toxicity scores per patient are listed in Table IV. Gastrointestinal toxicity was mild: only three patients had grade 3 diarrhoea and four mucositis; nausea and vomiting were not major problems. Alopecia was frequent but it was reversible in all patients after the end of treatment. The mean total dose of EPI administered was 450 mg $m^{-2}$ (range 225–525 mg $m^{-2}$) and no evidence of cardiac toxicity (WHO grade > 3) was recorded. With regard to bone marrow toxicity, the treatment led to a much greater suppression of leucocytes and platelets, as well as to a non-significant decrease in erythrocyte count. Myelosuppression tended to be cumulative, with lower and more prolonged nadirs after five cycles. Leucopenia (WHO grade 3 toxicity) affected only four patients. None of our patients required hospitalisation for sepsis, and the seven who experienced infections (mainly pulmonary) were all manageable on an outpatient basis.

### Discussion

Studies over the past few years seeking to define the role of post-surgical adjuvant chemotherapy in gastric cancer have yielded contradictory results (de Braud *et al.*, 1992; Atiq *et al.*, 1993; Hermans *et al.*, 1993), leaving the issue still unresolved. However, the modulation of 5-FU by folinic acid has led to the testing of promising new combinations in advanced gastric cancer (Murad *et al.*, 1993; Neri *et al.*, 1993).

Some preliminary data of ours (Neri *et al.*, 1992) had pointed to the ineffectiveness of post-surgical adjuvant chemotherapy in terms of disease-free interval and survival, for patients who at the time of surgery proved to be node negative. Since the presence of lymph node involvement is a highly unfavourable prognostic factor (Michelassi *et al.*, 1994), hence one requiring adjuvant treatment, we considered node-positive patients as those who stood to benefit the most

**Table IV** Toxicity according to World Health Organization grade

| | *Grade* 0 | 1 | 2 | 3 | 4 | *Incidence of grade 3 or 4 toxicity (%)* | |
|---|---|---|---|---|---|---|---|
| Emesis | 11 | 19 | 6 | – | – | – | – |
| Diarrhoea | 12 | 17 | 16 | 3 | – | 3/48 | (6.3) |
| Mucositis | 7 | 12 | 25 | 4 | – | 4/48 | (8.3) |
| Alopecia | 9 | 11 | 28 | – | – | – | – |
| Cardiac | 18 | 26 | 4 | – | – | – | – |
| Hepatic | 14 | 18 | 16 | – | – | – | – |
| Neurological | 23 | 25 | – | – | – | – | – |
| Renal | 24 | 20 | 4 | – | – | – | – |
| Anaemia | 17 | 19 | 11 | 1 | – | 1/48 | (2.1) |
| Leucopenia | 11 | 14 | 19 | 4 | – | 4/48 | (8.3) |
| Thrombopenia | 15 | 19 | 14 | – | – | – | – |

from post-surgical chemotherapy. This approach is in line with observations reported by other authors (The Gastrointestinal Tumor Study Group, 1982) who, with a therapeutic scheme different from ours, singled out patients with more advanced (T3–T4) gastric carcinomas as the ones likely to profit from adjuvant treatment, provided its dose intensity was high enough. At the same time, several investigators have focused on standardising the surgical techniques used in this pathology (Hermans *et al.*, 1993; Bunt *et al.*, 1994), since lymph node status plays a crucial role in the prognosis and choice of treatment. Moreover, starting from the observation that the majority of patients are diagnosed with stages III and IV gastric cancer, other researchers view preoperative chemotherapy as a more than promising approach to the integrated treatment of gastric cancer (Wilke *et al.*, 1989; Fink *et al.*, 1993; Rougier *et al.*, 1994), so much so that preoperative chemotherapy appears to be an attractive tool for clinical investigation in earlier stages of gastric cancer (Wils *et al.*, 1994).

In this study a survival advantage for patients treated with EPI–LV–5-FU was achieved and adjuvant chemotherapy was associated with a consistent delay in the appearance of recurrent or metastatic cancer. The treated patient group, moreover, had relatively fewer hepatic metastases than the controls, which, in agreement with Coombes *et al.* (1990) and Estapè *et al.* (1991), suggests a protective effect of adjuvant chemotherapy on blood-borne cancer dissemination. Acute toxicity was mild and treatment was well tolerated by patients, all of whom were treated on an outpatient basis. Long-term toxicity was non-existent and no case of cardiac toxicity was observed. We find these results sufficiently encouraging that, even before the completion of our 5 year follow-up, we have started using this adjuvant chemotherapy schedule with all node-positive gastric cancer patients. At the same time, we view as more promising candidates for adjuvant treatment patients earlier than stage III, that is, those with the lowest residual microscopic tumours after surgery. Furthermore, to optimise the chances of positive results we start treatment within 6 weeks after surgery and select a therapeutic schedule that produces a high degree of efficacy, with a grade of toxicity that is acceptable yet does not compromise the optimum dose intensity of treatment.

**Acknowledgements**
We wish to acknowledge the assistance of Dr Eda Berger in the translation and revision of this manuscript. This work was partially supported by the 'Associazione Toscana Cure e Ricerche Oncologiche'.

### References

ALEXANDER AR, KELSEN DP AND TEPPER JE. (1993). Cancer of the stomach. In *Cancer: Principles and Practice of Oncology*, DeVita VT, Hellman S and Rosenberg SA (eds) pp. 818–848. JB Lippincott: Philadelphia.

ATIQ OT, KELSEN DP, SHIU MH, SALTZ L, TONG W, NIEDZWIECKI D, TROCHANOWSKI B, LIN S, TOOMASI F AND BRENNAN M. (1993). Phase II trial of postoperative adjuvant intraperitoneal cisplatin and fluorouracil and systemic fluorouracil chemotherapy in patients with resected gastric cancer. *J. Clin. Oncol.*, **11**, 425–433.

BALIKDJIAN D, VAN HOUTTE P AND LUSTMAN-MARECHAL J. (1980). Place de la radiotherapie dans le traitement post operatoire des tumeurs de l'estomac. *Rev. Franc. Gastroenterol.*, **162**, 496–504.

BLEIBERG H, GERARD B AND DEGUIRAL P. (1992). Adjuvant therapy in resectable gastric cancer. *Br. J. Cancer*, **66**, 987–991.

BRUCKNER HW, CHESSER MR, WONG H AND MANDELI J. (1991). Folate biochemical modulation regimen for the treatment of gastric cancer. *J. Clin. Gastroenterol.*, **13**, 384–389.

BUNT AMG, HERMANS J, BOON MC, VAN DE VELDE CJH, SASAKO M, FLEUREN GJ AND BRUIJN JA. (1994). Evaluation of the extent of lymphadenectomy in a randomized trial of Western- versus Japanese-type surgery in gastric cancer. *J. Clin. Oncol.*, **12**, 417–422.

COOMBES RC, SCHEIN PS, CHILVERS CE, WILS J, BERETTA G, BLISS JM, RUTTEN A, AMADORI D, CORTES-FUNES H AND VILLAR-GRIMALT A. (1990). A randomized trial comparing adjuvant fluorouracil, doxorubicin and mitomycin with no treatment in operable gastric cancer. *J. Clin. Oncol.*, **8**, 1362–1368.

DE BRAUD F, BAJETTA E, DI BATOLOMEO M AND COLLEONI M. (1992). Adjuvant chemotherapy for cancer of gastrointestinal tract: a critical review. *Tumori*, **78**, 228–234.

DECARLI L AND LA VECCHIA C. (1988). Cancer mortality in Italy. *Tumori*, **74**, 6623–6632.

ENGSTROM PF, LAVIN PT, DOUGLASS HO AND BRUNNER KW. (1985). Post-operative adjuvant 5-fluorouracil plus methyl-CCNU therapy for gastric cancer patients. Eastern Cooperative Oncology Group Study. *Cancer*, **55**, 1868–1873.

ESTAPÈ J, GRAU JJ, LOCOBENDAS F, CURTO J, DANIELS M, VIGNOLAS N AND PERA C. (1991). Mitomycin C as an adjuvant treatment to resected gastric cancer. *Ann. Surg.*, **213**, 219–221.

FINK U, SCHUHMACKER C, BOTTCHER K, BUSH R, DITTLER HJ, HELMBERGER H, BARTELS H, STEIN HJ AND SIEWERT JR. (1993). Neoadjuvant chemotherapy with Etoposide/Adriamycin and Cisplatin (EAP) in locally advanced gastric carcinoma. In *Adjuvant Therapy of Cancer VII*, Salomon SE (ed) pp. 272–280. JB Lippincott: Philadelphia.

HAIM N, COHEN Y AND HONIGAM J. (1982). Treatment of advanced carcinoma with 5-fluorouracil, adriamycin and mitomycin. *Cancer Chemother. Pharmacol.*, **8**, 277–280.

HAVLIN KA AND MACDONALD JS. (1992). Gastric cancer: chemotherapy of advanced disease. In *Gastrointestinal Oncology*, Algren JD, Macdonald JS (eds) pp. 171–179. JB Lippincott: Philadelphia.

HERMANS J, BONENKAMP JJ, BOON MC, BUNT AMG, OHYAMA S, SASAKO M AND VAN DE VELDE GJH. (1993). Adjuvant therapy after curative resection for gastric cancer: Meta-analysis of randomised trials. *J. Clin. Oncol.*, **11**, 1441–1447.

INTERNATIONAL UNION GAINST CANCER. (1987). *Classification of Malignant Tumours*, Hermanek P and Sobin LH (eds). Springer: Geneva.

KAPLAN EL AND MEIER P. (1958). Non parametric estimation from incomplete observation. *J.Am. Stat. Assoc.*, **53**, 457–481.

KORNEK G, SCHULZ F, DEPISH D, ROSEN H, KWASNY W, SEBESTA C AND SCHEITHAUER W. (1992). A phase I–II study of epirubicin, 5-fluorouracil and leucovorin in advanced adenocarcinoma of the stomach. *Cancer*, **71**, 2177–2180.

MACDONALD JS, SCHEIN PS, WOOLLEY PV, SMYTHE T, UENO W AND HORT D. (1980). 5-Fluorouracil, doxorubicin and mitomycin (FAM) combination chemotherapy for advanced gastric cancer. *Ann. Intern. Med.*, **93**, 533–536.

MICHELASSI F, TAKANISHI DM, PANTALONE D, HART J, CHAPPELL R AND BLOCK GE. (1994). Analysis of clinicopathologic prognostic features in patients with gastric adenocarcinoma. *Surgery*, **116**, 804–810.

MILLER AB, HOOGSTRATEN B, STAQUET M AND WIKLER A. (1981). Reporting results of cancer treatment. *Cancer*, **47**, 207–214.

MURAD AM, SANTIAGO FF, PETROIANU A, ROCHA PR, RODRIGUES MA AND RAUSCH M. (1993). Modified therapy with 5-fluorouracil, doxorubicin, and methotrexate in advanced gastric cancer. *Cancer*, **72**, 37–41.

NERI B, GEMELLI MT, LOTTINI G, SAMBATARO S, FABBRONI S, LOTTINI L AND BRUNO S. (1992). Epidoxorubicin, high dose leucovorin and 5-Fluorouracil in advanced measurable gastric cancer: a phase II study. *Anticancer Res.*, **12** (suppl. 6A), 1927.

NERI B, GEMELLI MT, ANDREOLI F, BRUNO S, FABBRONI S, LEONE V, VALERI A AND BORRELLI D. (1993). Epidoxorubicin and high dose leucovorin plus 5-fluorouracil in advanced gastric cancer. *Anti-Cancer Drugs*, **4**, 323–326.

OGAWA M AND TAGUCHI T. (1990). Upper gastrointestinal tumors. In *Cancer Chemotherapy and Biological Response Modifiers*, Pinedo HM, Chabner BA and Longo DL (eds) pp. 456–459. Elsevier.

PREUSSER P, WILKE H, ACHTERRATH W, FINK U, LENAZ A, HEINICKE A, MEYER J AND BUENTE H. (1989). Phase I study of a combination of etoposide, doxorubicin and cisplatin in advanced measurable gastric cancer. *J.Clin. Oncol.*, **7**, 1310–1317.

ROUGIER P, LASSER P, DUCREUX M, MAHJOUBI M, BOGNEL C AND ELIAS D. (1994). Preoperative chemotherapy of locally advanced gastric cancer. *Ann. Oncol.*, **5**, 59–68.

SIMON R. (1985). Size of phase III cancer clinical trials. *Cancer Treat. Rep.*, **69**, 1087–1093.

SAS INSTITUTE INC. (1987). SAS/STAT Guide for Personal Computers, Version 6 edn. SAS Institute: Cary, NC.

THE GASTROINTESTINAL TUMOR STUDY GROUP. (1982). Controlled trial of adjuvant chemotherapy following curative resection for gastric cancer. *Cancer*, **49**, 1116–1122.

WAXMAN ASJ. (1992). Chemotherapy for gastric cancer. *Gut*, **33**, 1153–1154.

WILKE H, PREUSSER P, FINK U, GUNZER U, MEYER HJ, SIEWERT JR, ACHTERRATH W, LENAZ L, KNIPP H AND SCHMOLL HJ. (1989). Preoperative chemotherapy in locally advanced and nonresectable gastric cancer: a phase II study with Etoposide, Doxorubicin and Cisplatin. *J. Clin. Oncol.*, **7**, 1318–1326.

WILS J, MEYER HJ AND WILKE H. (1994). Current status and future directions in the treatment of localized gastric cancer. *Ann. Oncol.*, **5**, 69–72.